# Animal Source Food Consumption in Young Children from Four Regions of Ethiopia: Association with Religion, Livelihood, and Participation in the Productive Safety Net Program

**DOI:** 10.3390/nu11020354

**Published:** 2019-02-08

**Authors:** Kaitlin S. Potts, Afework Mulugeta, Alessandra N. Bazzano

**Affiliations:** 1 Department of Global Community Health and Behavioral Sciences, Tulane University School of Public Health and Tropical Medicine, New Orleans, LA 70112, USA; kstorck@tulane.edu; 2 Department of Epidemiology, Tulane University School of Public Health and Tropical Medicine, New Orleans, LA 70112, USA; 3 Department of Nutrition and Dietetics, School of Public Health, College of Health Sciences, Mekelle University, Mekelle 231, Ethiopia; afework.mulugeta@gmail.com

**Keywords:** child nutrition disorders, animal source foods, diet, food and nutrition, dietary diversity, food assistance

## Abstract

Introduction: Child undernutrition remains a challenge globally and in the geographically diverse country of Ethiopia. Improving dietary diversity and consumption of animal source foods are important for improving child nutrition and corresponding health outcomes. Objective: The objective of the study was to identify household and community factors associated with consumption of animal source foods among 6 to 36-month-old children from four regions of Ethiopia. Methods: A cross-sectional survey using multistage probability sampling in eight geographic zones and four regions of Ethiopia took place in 2015 with parents/caretakers of 6 to 36-month-old children. Data was collected on demographic information, proxy indicators of socioeconomic status, and food consumed by the child the day before the survey. Results: Increased child age, pastoral livelihood, Muslim religion, and participation in the Productive Safety Net Program were associated with increased consumption of animal source foods. Odds of animal source foods consumption increased by 8% with each 3-month age increase. Children from pastoralist households were the most likely to have consumed animal source foods in the preceding 24 hours as compared with those in agro-pastoralist households (0.21 times as likely) or those in agriculturalist/farming households (0.15 times as likely). The odds of consumption of animal source foods for families with food aid or safety net support was 1.7 times greater among those receiving traditional support from the Productive Safety Net Program and 4.5 times greater for those in the direct support arm of the program. Conclusions: The findings illustrate the importance of accounting for local context and community characteristics, such as livelihood and religion, when undertaking programming designed to improve diversity of children’s diets through increasing animal source foods. In addition, the Productive Safety Net Program may be a critical determinant of dietary diversity for young children in these regions.

## 1. Introduction

Child undernutrition remains a challenge worldwide. In 2017, over 20% of the world’s children under the age of five were stunted and 7.5% suffered from wasting [1] Maternal and child undernutrition is estimated as the largest underlying risk factor of mortality and morbidity for children less than five worldwide [2]. Estimates have identified undernutrition as the underlying cause of up to 45% of under-five deaths [3]. In addition to impaired physical and cognitive development, undernutrition in childhood is linked to poor reproductive outcomes, metabolic and cardiovascular health issues, and diminished economic prospects later in life [2,4,5]. Addressing the challenge of child undernutrition has long been considered an urgent priority and remains at the top of the world’s agenda, as noted by the second United Nation’s Sustainable Development Goal, “Zero Hunger” [6].

Ethiopia has made great strides in improving the nutritional and health status of children over recent decades. Child stunting in the country has decreased by 20 percentage points since 2000, but 38% of children under five remain stunted as of 2016, with 10% wasted, suffering from chronic and acute hunger and food insecurity [7]. This varies greatly by region with 46% of under-five children stunted in Amhara and nearly 18% wasted in Afar [7]. The economic cost is high, and it is estimated that Ethiopia annually incurs 4.7 billion USD in losses to health, education, and productivity, or 16.5% of GDP, due to child undernutrition [8].

Following recommended standards for feeding is essential for improving nutritional outcomes for children [9]. Complementary feeding in addition to continued breastfeeding beginning at six months of age is necessary for continued and adequate physical and cognitive growth in young children [10,11,12]. Sufficient quantity and diversity of high-quality foods is essential for the provision of adequate micro- and macronutrients to the child [13]. Dietary diversity has been shown to contribute positively to nutritional status [11]. Animal source foods (ASF) including meats, eggs, and dairy are an excellent source of protein, calories, and necessary micronutrients and have been linked to improved nutritional status in children, including reduced stunting [14,15,16,17]. In addition to anthropometric benefits, a systematic review reported that consuming ASF, including milk, is linked to improved cognitive function among undernourished children in low-income settings [18]. ASF comprise three of the seven food groups used to determine sufficient dietary diversity in children, as measured by the World Health Organization’s (WHO) indicators of infant and young child feeding (IYCF) status [19]. This acted as the conceptual framework for identifying the importance of ASF to child nutritional status. Similar to dietary diversity, ASF consumption is low among Ethiopian children [20,21,22]. 

Access to and availability of ASF is often limited for many children in resource poor and low and middle-income settings due to higher costs. Food insecurity has been associated with child undernutrition in Ethiopia and other countries [23], and nutritional status also differs among livelihoods, especially comparing pastoralists to other livelihood groups, in this part of Africa [24]. Availability of ASF to children may also be impacted by other factors such as local culture and religion. 

Achieving optimal dietary diversity for children in Ethiopia has been a challenge until fairly recently due to economic and geographic factors. In 2011, a staggeringly low 4% of children aged 6 to 23 months met the recommendation for dietary diversity [25]; while this has improved, still only 14% were receiving adequately diverse diets in 2016 [7], with as few as 2.6% of children from the Afar region achieving this. Given the definition of adequate dietary diversity, low consumption of ASF is an important contributor—only 8% of breastfed children aged 6 to 23 months reported consumption of meat, fish, or poultry, and only 17% reported egg consumption in the 24 hours prior to the interview for the 2016 EDHS [7]. 

Approaches to address child undernutrition with improved complementary feeding and dietary diversity include nutrition education through behavior change communication strategies, as well as provision of additional complementary foods [26,27]. Both approaches have been effective in improving the nutritional status of children in food insecure areas, but given the multifaceted nature of undernutrition, multiple strategies operating simultaneously are needed to make sustained improvements [9]. A systematic review of complementary feeding interventions illustrated their effectiveness at improving nutritional status, especially when educational components were combined with provision of food [27]. 

Ethiopia has demonstrated commitment to tackling the problem of undernutrition with its Food and Nutrition Policy [28], National Nutrition Program [29], Nutrition Sensitive Agriculture Strategy [30], and Seqota Declaration [31] released in 2017 with the goal of eliminating child undernutrition by 2030. The Community Based Nutrition Program (CBN), the Targeted Supplementary Feeding Program, and the Productive Safety Net Program (PSNP) in Ethiopia combine to offer evidence-based interventions for the improvement of infant and young child feeding, including dietary diversity [29,32]. Evaluation of the CBN program has shown positive impact on child malnutrition [33]. Additionally, exposure to these programs in Ethiopia has been associated with improved complementary feeding, including intake of ASF [34]. 

While much research has looked at dietary diversity in Ethiopian children, there has been less focus on identifying factors associated with consumption of ASF in children. Minimal research has attempted to identify how individual characteristics relate to the feeding of children. Ethiopia is a large and diverse country of almost 100 million people with different cultures, languages, livelihoods, and religions [35]. The diversity inherent in the Ethiopian population may impact child feeding and nutrition, but limited research (predominantly from the grey literature) has looked into this [36,37]. Using formative research to identify cultural factors related to optimal child feeding improves the effectiveness of interventions [38], thus more research in this area is warranted.

The present study aimed to address a gap in the literature relating to ASF consumption among children in diverse settings of rural Ethiopia. The objective of the present study was to determine factors associated with ASF consumption among 6 to 36-month-old children in the study areas from Ethiopia. 

## 2. Methods

### 2.1. Study Setting

The study was conducted in eight zones and four regions in Ethiopia: Afar, Amhara, Benishangul-Gumuz, and Tigray from October through December 2015. Geographic remoteness and resource limitations necessitated limiting the survey to two zones per region. The zones were purposively selected based on IYCF factors identified from previous research to capture a range of experiences within the region [39]. The eight zones surveyed were: Zone 1 and 4 in Afar, South Wollo and West Gojjam in Amhara, Asosa and Metekel in Benishangul-Gumuz, and Eastern and North Western Zones in Tigray. The Afar region in the northeastern part of Ethiopia is a predominantly pastoralist region. Afar is a region where the highest burden of underweight (36%) and second highest burden of wasting (18%) in under-five children was reported [7]. Amhara, Benishangul-Gumuz, and Tigray are largely agrarian regions. The Amhara region is characterized by the highest prevalence of stunting in Ethiopia where 46% of the under-five children in the region are stunted [7]. The second highest prevalence of stunting among under-five children is reported from Benishangul-Gumuz [7]. About 39%, 23%, and 11% of the under-five children from Tigray are stunted, underweight, and wasted, respectively [7]. The heavy burden of child malnutrition, low levels of child dietary diversity, and population diversity of these regions present an optimal area for investigating the research question.

### 2.2. Study Design

The data in the present study were collected for operation purposes by an international non-governmental organization and analyzed separately by the study authors as strictly de-identified. A cross-sectional study design was employed to conduct the study on ASF consumption in 6 to 36- month-old children from the four regions of Ethiopia. The inclusion criteria included children of 0 to 36 months of age living in the households of interviewed mothers/caretakers in rural areas. Because the outcome of interest (ASF) should not be given to children younger than 6 months of age based on international recommendations, this analysis excluded children in the sample younger than 6 months of age, but the remaining children were still the youngest in the household since only one child per household was included.

### 2.3. Sample Size

A multi-stage sampling technique was used to select study participants. The sample size was calculated to detect a 10% change at household level in receipt of nutrition services across the sampled zones with the anticipation of a follow up survey. One hundred simulations were conducted to determine the sample size using the R package clusterPower [40] with 80% power and type-1 error rate of 5%. Between-cluster variance was assumed to be 0.1 in all survey rounds and sample size simulations. The results indicated that sampling of ten clusters per zone with fifteen households per cluster would allow equal probability of selection for all households that met the inclusion criteria per zone. Minimum samples of 143 households per zone were indicated to detect the identified change. In order to preserve internal validity, 12 clusters per zone were used so that the minimum sample sizes could be met without the need for replacement of non-responsive households or unreachable clusters. These methods were employed to eliminate sampling bias as much as possible. Although the initial sample size was calculated for a different outcome, the sample of 1009 was amply large to detect the hypothesized differences in the present study. Using G*Power [41], a 15 percentage point difference in consumption of animal source foods across three groups, similar to the religion variable, is detectible using a contingency table chi square test for independence at 80% power and 5% type-1 error rate with a sample of 531.

### 2.4. Sampling

As required by the local government partner, the Ethiopian Central Statistical Authority (CSA) used probability proportion to size random sampling to select twelve clusters in each zone from the 2007 census listing of rural enumeration areas. These were sampled without replacement if the cluster was deemed unreachable during data collection due to unfavorable field conditions. Upon arrival in each cluster, the survey team consulted community leaders (e.g., health extension workers) to define the geographical confines of the cluster, which corresponded to a local village. The last stage of sampling began with the full enumeration of the cluster to develop a listing of all eligible households (those with 0–36-month-old children) in the area. The survey team leader then used a random number table to randomly sample fifteen households. Enumerators were instructed to revisit sampled households three times before it was considered non-responsive. Each household completed only one household level survey, based on the youngest child living in the house, so the household survey gives data on one child/caregiver dyad.

### 2.5. Data Collection Methods and Tools

The household survey questionnaire included sections on demographics, water and sanitation, reproduction, breastfeeding, complementary feeding, nutrition services, maternal and child health, and food security. The questionnaires were developed in English, translated to Amharic for Amhara, Afar, and Benishangul-Gumuz regions and Tigrigna for Tigray, back translated to English, and pre-tested. Experienced enumerators who spoke the languages of the surveyed areas implemented the study and were trained on the survey protocol and tools during a four-day training session held in Addis Ababa prior to data collection. The enumerators conducted the household surveys in the homes of caregiver participants. Paper questionnaires were used for data collection. The completed questionnaires were then entered into a database by separate data entry persons and converted into excel spreadsheets.

### 2.6. Ethics

Verbal informed consent was obtained from all participants prior to data collection during fieldwork. The authors received approval from the Tulane University Health Sciences Institutional Review Board for secondary analysis of data collected by a non-governmental agency. 

### 2.7. Measures

#### 2.7.1. Outcomes

The primary outcome measure for the present analysis was consumption of ASF by 6–36-month-old children. The questionnaire asked parents/caretakers what types of foods the child had eaten in the 24 hours prior to the survey. These questions were matched to those used in the 2011 EDHS [25], and the respondent stated if the child consumed any amount of foods from a predefined list. The animal source foods in the list included eggs, fish, yogurt, cheese, milk, meat (including beef, poultry, pork, lamb, and any other meat not mentioned), and organ meats (e.g., liver). 

As a secondary outcome of interest, minimum dietary diversity (MDD) was calculated to identify if a positive relationship between ASF and MDD was present in this sample. MDD was defined according to the WHO definitions of IYCF indicators [19], requiring a minimum consumption of four out of seven pre-defined food groups in the previous 24 hours for the child’s diet to be considered adequately diverse. The seven food groups include: grains, roots, and tubers; legumes and nuts; dairy products (milk, yogurt, cheese); flesh foods (meat, fish, poultry, and liver/organ meat); eggs; vitamin-A rich fruits and vegetables; and other fruits and vegetables [19]. Since three of the seven food groups are ASF, it was anticipated that ASF consumption contributed to dietary diversity in the sample. 

A group of cases from the region of Benishangul-Gumuz were identified to have answered the dietary recall questions in a way that did not meet the requirements of the study protocol. This resulted in the need to exclude 50 participants to eliminate introducing bias in the measurement of the outcome of interest.

#### 2.7.2. Independent Variables

Measured independent variables were selected based on their hypothesized influence on consumption of ASF. Child and household characteristics included in this analysis were child sex and age; respondent age, education, and occupation; and household total number of children, assets, livelihood, religion, and livestock ownership. 

Education is a dichotomous variable identifying caretakers reporting any versus no formal or informal (e.g., religious schooling that includes learning to read) education. Respondent occupation was reduced to a three-level variable to include farmer, housewife (defined as having no employment outside the home), and other occupations. Household asset ownership was determined from a set of twelve assets (equivalent to those asked on the EDHS) including electricity, a watch or clock, a radio, a television, a mobile telephone, a non-mobile telephone, a refrigerator, a table, a chair, a bed with a mattress (cotton/sponge/spring), an electric mitad (a grill or cooktop used for preparing injera or bread), and a kerosene lamp/pressure lamp. Ownership of these assets were summed to assign a score of asset ownership to households. Family livelihood was determined from three questions regarding activities of anyone in the household: ownership of agricultural land, use of land to grow crops to consume or sell, and movement for the purpose of obtaining food or water for livestock. Households were categorized as agriculturalists if they owned land and used it to grow crops for consumption or to sell but did not move in search of food or water for livestock. Participants that grew crops for sale or consumption on their land and moved in search of food and water for livestock were categorized as agro-pastoralists. Households moving in search of food or water for livestock, but not owning land used to grow crops for sale or consumption, were classified as pastoralist households. Religion was constructed as a three-level variable to identify Ethiopian Orthodox Christians, Muslims, and other religions (including other Christian faiths). Livestock ownership was assessed as the total number of animals owned.

Occupation and livelihood were highly correlated (Pearson correlation coefficient = 0.650) since the farmer occupation and agriculturalist livelihood captured almost the same group. Thus, these were combined to eliminate issues of multi-collinearity in the multivariable logistic regression model, and allowed for fewer missing cases. Participants/caregivers who reported their occupation as housewife or other but were assigned to pastoralist or agro-pastoralist livelihoods were analyzed as pastoralist or agro-pastoralist, not as housewives or other occupation. The resulting variable was considered as the household-level livelihood/occupation. 

Food security was assessed using a portion of the Household Food Insecurity Access Scale (HFIAS) developed by the Food and Nutrition Technical Assistance Project in 2007 [42]. Time limitations prevented the full set of HFIAS questions from being included on the questionnaire. The questions used were intended to measure insufficient food intake and its physical consequences, defining severe food insecurity by the HFIAS [43]. These asked about the occurrence (yes/no) and frequency (rarely, sometimes, often) during the previous month of the following food insecure events: was there ever no food in the household, did they ever go to bed hungry because there was not enough food to eat, and did they ever go a whole day and night without eating because they did not have enough food. A severe food insecurity score was calculated per the full HFIAS questionnaire [42], by summing the frequency of occurrence (0 for never, 1 for rarely, 2 for sometimes, and 3 for often) of each event, giving a score ranging from 0, indicating the household had not experienced any severe form of food insecurity, to 9, indicating all three severe food insecurity events happened often.

Participants were asked about receipt of food aid and participation in the PSNP during the previous year. The PSNP provides food, cash, or a combination in exchange for labor on public works projects supported by the Agriculture Extension Program [32]. Direct support from the PSNP where no labor is required was also evaluated. Pregnant and lactating women, and those with children suffering from acute malnutrition, qualify for temporary direct support from the PSNP with some conditionalities of antenatal care and nutrition education.

### 2.8. Analysis

Data was transferred into SPSS Statistics, version 24 (IBM Corp, Armonk, NY, USA) for analysis. Descriptive characteristics are given as proportions (%) or means for categorical and continuous variables, respectively. Bivariate analysis of ASF consumption with independent variables was conducted using Pearson’s Chi Square test of independence for categorical variables, and independent samples t-test and Wilcoxan rank-sum test for continuous variables.

Multivariable logistic regression was used to calculate adjusted odds ratios (OR) and 95% confidence intervals (CI). Two adjusted models are presented. The first is adjusted for age to remove the effect of increased consumption of foods, including ASF, resulting from potential age differences between groups. The second adjusted model includes age, livelihood, religion, and the food aid/safety net variable. The model building strategy was similar to that outlined by Hosmer and Lemeshow [44]. The initial selection of variables was based on a priori thought of their relation to the outcome and their availability in the data. The initial multivariable model included all effects that were associated with the outcome in unadjusted analysis at *p* < 0.25, then those that were not significantly improving the model (*p* > 0.05 based on likelihood ratio tests) were removed until a final parsimonious model was reached with stable parameter estimates. Variables that had unadjusted significant associations with ASF but had been removed were re-entered into the final model to check again for significant contribution in the smaller model. None were retained at this point. Child sex was not included in the final model since it was not associated with ASF consumption, but when it was included, it did not alter the results. Interactions among the variables in the final model were not tested due to the small cell sizes that would result. The final model did not show indication of poor fit, based on Hosmer and Lemeshow’s test for goodness of fit using deciles (*p* = 0.6736) [44]. All adjusted ORs from the final model are presented regardless of statistical significance.

Missing values were limited for the majority of variables used and were excluded from analyses that used those variables but were not excluded across all other analyses in order to preserve sample size. Imputation was not used for multiple logistic regression.

## 3. Results

A total of 1299 households were surveyed across 8 zones from 88 clusters, of which 10 children had missing age and 230 were younger than 6 months and were thus excluded from analysis. Among the 1059 remaining, 50 were excluded cases with compromised dietary recall questions. Finally, a sample size of 1009 was used for analysis. 

### 3.1. Participant Characteristics

Among the 1009 children in the sample, 53% were male and were on average 19.6 months of age (Table 1). The average age of parents/caregivers was 29 years and most (97%) said they were the mothers of the index children and one-third reported having attained any formal education. The surveyed households predominantly belonged to two religious groups, Ethiopian Orthodox and Islam at 41% and 54%, respectively. The sampled households had only 1.4 assets (out of potential 8) on average. Pastoralists comprised 20%, 29% reported both pastoral and agricultural activities, and 42% were agriculturalists (farmers). Those respondents who did not fall into one of these livelihood categories reported being housewives (3.5%) or had other occupations (5.8%). Households owned an average of 11 animals. The average severe food insecurity score was 0.7, and the majority (62%) said they had not received any food aid, nor had they participated in the PSNP in the past year. About 11% said they had received non-PSNP related food aid, 20% said they had participated in the PSNP (not direct support), and 8% said they had been the beneficiary of PSNP direct support during the past year (Table 1). 

### 3.2. ASF and MDD

**Figure 1:**
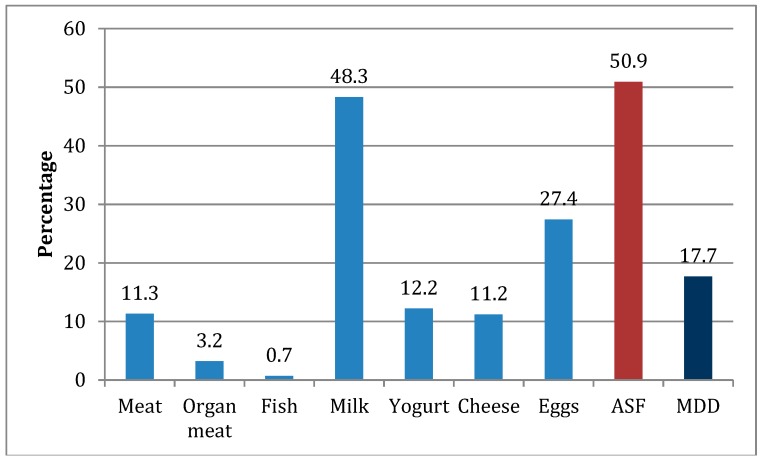
Proportion of sample 6–36-month-old children consuming animal source foods and meeting the minimum dietary diversity. ASF, animal source foods; MDD, minimum dietary diversity.

The recommended minimum dietary diversity of four or more different types of foods the previous day was met by 18% of the surveyed children and 51% had consumed ASF (Table 1). The most commonly consumed ASF was milk (48%), whereas meat was consumed by 11% of children on the day before the survey (Figure 1). Less than 1% of the children had eaten fish, but the study took place in predominantly non-fishing communities. Only 3% had eaten organ meats such as liver. The number of those having consumed yogurt and cheese in the previous 24 hours was 12% and 11%, respectively. 

Those children who consumed ASF during the previous day were more likely to meet the recommended minimum dietary diversity. Of those who had eaten ASF, 30% met the minimum dietary diversity compared to only 4% of those who had not consumed ASF. 

### 3.3. Unadjusted Associations

Increased ASF consumption was associated with increasing child age, household assets, religion, livelihood, livestock ownership, severe food insecurity score, and food aid or safety net participation (Table 1). The unadjusted relationship between ASF and severe food insecurity score was not in the anticipated direction; those with a higher score indicating more severe food insecurity experiences had consumed more ASF. This was likely influenced by the inability of the severe food insecurity score to capture mild and moderate forms of food insecurity, and the inclusion of milk in ASF. The direction of the unadjusted associations between ASF and child age, household assets, and livestock ownership were in the expected directions as older children eat more foods, increased household assets may be a proxy for improved socioeconomic status (SES), and increased livestock ownership may indicate higher SES as well as improved access to some ASF including milk. The distribution of household asset ownership differed by ASF consumption as shown by the significant Wilcoxan rank-sum test, despite the median (Q1, Q3) being the same across groups. The mean number of assets owned was greater in the group consuming ASF. Child sex, total number of children, and respondent education were not associated with ASF consumption, while caregiver’s age had a moderate association with ASF in the positive direction (Table 1). 

Muslim children had consumed ASF (60% of Muslim children) at a higher rate than Orthodox children (40%) and those from other religions (39%, Table 1). Among the different livelihood and occupation groups, more children of pastoralist households had eaten ASF the previous day (85% of pastoralists) than agro-pastoralists (47%) and agriculturalists/farmers (36%). Children from households receiving food aid or participating in the PSNP consumed more ASF than children in households without these types of assistance (Table 1).

### 3.4. Adjusted Associations

In the adjusted model, associations with ASF were retained with child age, religion, livelihood/occupation, and food aid/PSNP participation (Table 2). The livelihood/occupation groups of “housewife” and other were combined during the multivariable analysis given the small numbers of people in them. Household assets and livestock ownership were no longer significantly associated with ASF consumption when adjusted for these other variables, so they were not included in the final model. 

Children from Orthodox households were 30% less likely to have consumed ASF the day prior to the survey than children from Muslim households (Table 2). Compared with children of pastoralist livelihood families, those from agro-pastoralist households were 79% less likely to have eaten ASF, children from agriculturalist families were 85% less likely, and those with housewife and other occupations were 68% less likely compared to the pastoral families. Finally, those receiving non-PSNP related food aid were not significantly more likely to have consumed ASF based on the adjusted model, but those who had participated in the PSNP during the past year were. Children from families who had participated in the PSNP as regular participants (doing public works) were 74% more likely, and those who had participated in the PSNP as direct support recipients were 4.47 times as likely, to have consumed ASF the day before the survey, compared to those who did not receive any food aid and did not participate in the PSNP during the previous year (Table 2). 

## 4. Discussion

Dietary diversity was adequate in less than 20% of the children in this study and only around half had consumed any ASF, including milk, milk products, and eggs in the 24 h preceding the interview. The results confirmed the hypotheses that ASF consumption among children in the rural areas studied in Ethiopia is associated with certain household and community characteristics, including religion and livelihood. The factors associated with the consumption of ASF in 6–36-month-old children from the four regions of Ethiopia were child age, religion, livelihood/occupation, and participation in the safety net program. Children of Ethiopian Orthodox households were less likely to have consumed ASF than children from Muslim households. Pastoralist children were more likely to consume ASF compared to children from agricultural households or other livelihoods. Additionally, children in households with PSNP support, whether by traditional participation or as direct support recipients, consumed more ASF than children in households without such support.

Household assets and livestock ownership were not significantly associated with ASF consumption in adjusted analyses. The very low level, only 1.4 assets per household out of 8 on average, and lack of variation in household asset ownership may partly explain this finding. Similarly, measurement of livestock ownership was not able to account for animal type. This could impact the results and may have contributed to the non-significance in adjusted analyses. Different categorization of livestock numbers was explored but did not lead to differential results, and it was decided to avoid further assessment to prevent bias introduced by looking for the best cutoff point or categorization to enhance statistically significant findings. 

The finding that consumption of ASF increases with age is not surprising given both improved feeding skills and increased caloric needs of children in this age range. Since the study design did not include a method to measure total energy intake, it was not possible to control for this during analysis. However, previous research has indicated that parents and community members do not feel young children are ready for meats and other non-dairy animal products often until later ages [37,45].

Ethiopian Orthodox Church doctrine includes fasting days throughout the year, including most Wednesdays and Fridays, and extended periods of the Great Lent Fast (Hudaddie Tsom) of 55 days and Fast of the Prophets (Tsome Nebiyat) of 43 days when adults abstain from ASF including eggs and dairy [46]. This may impact children’s consumption of ASF, even if children are not required to fast, due to limited availability of ASF during extended fasting times. Caregivers who are fasting may be hesitant to prepare non-fasting foods for children out of fear of contamination. A recent study among Orthodox Christian households in Ethiopia found lower dietary diversity in children whose mothers reported that they did not want to serve ASF to their children while adults in the household were fasting due to fear of contamination [47]. A recent unpublished study reported by Alive & Thrive in Amhara during the extended Lenten fasting period found fear of contamination of family foods to be a significant barrier to optimal IYCF and feeding ASF to children in Orthodox families [48]. Authors reported partnering with religious leaders via cascade training to improve knowledge regarding the importance of ASF for children’s diets. 

Reduced presence of ASF in children’s diets during fasting times may not negatively impact health status as vegetarian diets have demonstrated to be healthful alternatives even for children. This has also been shown in the regions studied. A study by Gashu et al. found low prevalence of iron-deficiency anemia in school children in the Amhara region, Ethiopia, despite a predominantly plant-based diet [21]. Similarly, an Alive & Thrive report assessed religion and infant and young child feeding through analysis of the 2005 and 2011 EDHS and found Orthodox children were more likely to consume iron rich foods, yet less likely to meet the dietary diversity recommendations or consume ASF than children of other religions, confirming the findings of this study [49]. 

By contrast, Muslims engage in fasting for only a one-month period, Ramadan. A crucial difference is that consumption of ASF is permissible by Muslims during the evening meal throughout the month of Ramadan. The dates of data collection for this study did not include the month of Ramadan. 

The hypothesis that pastoralist livelihood positively contributes to ASF consumption is based on increased access to fresh milk, and at times meat, for children of livestock herders. Reports have estimated that pastoralists in some parts of Ethiopia may consume between 20% and 50% of dietary energy from milk and animal products, and that young children are especially reliant on the milk sources and are prioritized for consuming available milk [50,51,52]. Formative research conducted by the Milk Matters program among Ethiopian pastoralists in 2009 identified a strongly perceived benefit of animal milk among pastoralists and belief in its importance for preventing child malnutrition [36]. This could be protective for pastoralist children during fasting, even if they are in Orthodox communities. Evaluation of the Milk Matters intervention found that supporting milking animals resulted in increased milk availability and consumption among children, and stabilized nutritional status during the dry season [53]. Heavy reliance on milk and ASF for children in pastoralist communities increases vulnerability to dry seasons when availability from livestock decreases, emphasizing the seasonality of the food source and livelihoods [24,51,54]. However, other evidence suggests that the diets of pastoralist children remain inadequate despite higher milk consumption. Mengistu found low consumption of ASF other than milk in pastoralist communities in Ethiopia [55], coupled with inadequate energy intake, multiple micronutrient deficiencies, and a high prevalence of stunting. The current study similarly found that only 11% of participants had consumed flesh foods the day before the study, compared to 48% having had milk. 

Increased ASF consumption in this study among children of PSNP-supported households indicates the potential positive impact of food and cash transfers, coupled with nutrition counseling on child feeding. Cash transfers may free up resources for acquisition of nutritionally valuable foods including ASF [56,57,58,59]. Low household income has been associated with low dietary diversity in children in a recent study among Orthodox Christian households in North West Ethiopia [47]. There is evidence that access to the PSNP has increased household average caloric intake [60] and child dietary diversity and nutritional status [39,61,62]. One recent study challenged the effectiveness of the program at improving dietary diversity and nutritional status, using an econometric approach to data from the Ethiopian Socioeconomic Survey, however, the methods were not equivalent to those used in this study, nor to any others, limiting comparability [63]. 

### Strengths and Limitations

This study had several strengths, including that sampling resulted in equal probability of selection for households that met the inclusion criteria within the study areas and internal validity of the resultant large sample size. The diversity of sample population allowed for adequate proportions within the sample to test the main hypotheses and draw inference in rural Ethiopia. The careful modeling strategy allowed the most important effects to emerge in a parsimonious model giving adjusted ORs to understand the findings.

Limitations included: exclusion of participants due to errors during data collection, lack of comprehensive measures of socioeconomic status, and difficulty in interpreting food insecurity due to limited scope of questions included in the survey. The majority of households reported no severe food insecure events in the past 30 days despite the onset of a severe drought that had begun in 2015, so it is possible that some participants were experiencing mild to moderate food insecurity. However, the reporting of food aid and safety net participation may have compensated for this. The inability to ascertain the intensity, for example total number of months, and timing of food aid receipt is another limitation. However, defining this as any aid during the past year minimizes the risk of missing recipients who recently ended participation, when the effects may be ongoing. An additional limitation is the livestock ownership indicator, which did not distinguish between types of animals limiting its ability to proxy SES and identify increased access to ASF through household animal ownership. Finally, the cross-sectional design also limits the study’s findings. 

This study did not assess different aspects of the outcome such as total daily amount and type of ASF consumption and could not explore the typical frequency of intake. Exploration of predictors of different aspects of ASF intake amongst children may give different results.

Nonetheless, findings are likely representative of patterns in rural areas of the four regions in which the study took place. Generalizability beyond these regions in Ethiopia may be more limited, but further research in other regions would support the reliability of these results. Additional research into the associations between religion, livelihood, and PSNP participation with consumption of ASF while adequately controlling for socioeconomic factors and food security would improve upon these findings. Studies of the effectiveness of the PSNP to improve dietary diversity, including consumption of ASF, and nutritional status of children with proper evaluation designs should be pursued. 

These results imply the need to account for specific community characteristics such as religion and livelihood in interventions. Methods to maximize the quality of children’s diets with vegetarian-based fasting foods should be further explored in fasting communities and potentially incorporated into program messaging. Behavior change related to ASF for children among households during fasting periods may have limited effect unless issues such as contamination and children’s exemptions from fasting are highlighted. Engagement of religious leaders is likely to be beneficial. Pastoralist communities may not need to increase consumption of ASF, especially milk, but may lack access to other nutritionally diverse foods. It is essential to protect pastoral livestock in times of drought in order to maintain this essential food source for children. Livestock resiliency piloted by the Milk Matters program provides a useful strategy [53]. Participation in the PSNP appears to benefit children in terms of consumption of ASF and should be expanded to additional geographic areas and households in need, while continuing to evaluate its effectiveness at improving dietary and nutritional status outcomes in children of food insecure households. 

Household, family, and community characteristics play an important role in child feeding habits and norms. This study found that in addition to child age, the family’s religion, livelihood/occupation, and participation in the PSNP were significantly associated with child consumption of ASF. Such household and community factors should be considered when designing and targeting programs to improve child dietary diversity, nutritional status, and health.

## Figures and Tables

**Table 1:** Socio-demographic, economic characteristics, and animal source food consumption of the study participants from the four regions of Ethiopia, 2015 (*n* = 1009).

	ASF Consumption			
Variable (Missing)	No	Yes		Total
	*n* (row %) ^a^	*n* (row %) ^a^	*p*-Value	*n* (col %) ^a^
**Child Characteristics**				
Sex of child (6)			0.6995 ^b^	
Male	257 (48.7)	271 (51.3)		528 (52.6)
Female	237 (49.9)	238 (50.1)		475 (47.4)
Age of child in months (0)				
Mean (SD)	18.6 (8.84)	20.7 (8.45)	0.0001 ^c^	19.6 (8.7)
Median (Q1, Q3)	17 (10, 25)	21 (13, 27)		20 (12, 26)
**Caretaker and Household Characteristics**				
Respondent age in years (39)				
Mean (SD)	29.0 (6.3)	29.6 (6.3)	0.0979 ^c^	29.3 (6.3)
Median (Q1, Q3)	28 (25, 32)	30 (25, 34)		29 (25, 33)
Total number of children (168)				
Mean (SD)	4.0 (1.9)	3.9 (2.0)	0.8391 ^c^	4.0 (1.9)
Median (Q1, Q3)	4 (3, 5)	4 (2.5, 5)	0.5721 ^d^	4 (3, 5)
Respondent Education (8)			0.2367 ^b^	
No education	322 (47.9)	351 (52.2)		673 (67.2)
Any education	170 (51.8)	158 (48.2)		328 (32.8)
Number of household assets (29)				
Mean (SD)	1.3 (1.4)	1.5 (1.6)	0.0261 ^c^	1.4 (1.5)
Median (Q1, Q3)	1 (0, 2)	1 (0, 2)	0.0395 ^d^	1 (0, 2)
0	186 (51.1)	178 (48.9)	0.0336 ^b^	364 (37.1)
1	135 (53.2)	119 (46.9)		254 (25.9)
2–4	137 (43.8)	176 (56.2)		313 (31.9)
5–8	18 (36.7)	31 (63.3)		49 (5.0)
Religion (3)			<0.0001 ^b^	
Muslim	220 (40.2)	327 (59.8)		547 (54.4)
Orthodox	246 (59.6)	167 (40.4)		413 (41.1)
Other ^e^	28 (60.9)	18 (39.1)		46 (4.6)
Livelihood/Occupation (0)			<0.0001 ^b^	
Pastoral	30 (15.2)	167 (84.8)		197 (19.5)
Agro-pastoral	155 (53.1)	137 (46.9)		292 (28.9)
Agricultural/Farmer	272 (63.9)	154 (36.2)		426 (42.2)
Housewife	16 (45.7)	19 (54.3)		35 (3.5)
Other ^f^	22 (37.3)	37 (62.7)		59 (5.8)
Livestock ownership: number of animals owned (23)				
Mean (SD)	6.2 (22.4)	16.2 (27.5)	<0.0001 ^c^	11.3 (25.7)
Median (Q1, Q3)	1 (1, 6)	7 (1, 18)	<0.0001 ^d^	3 (1, 12)
0	47 (59.5)	32 (40.5)	<0.0001 ^b^	79 (8.0)
1–4	279 (60.8)	180 (39.2)		459 (46.6)
5–10	70 (48.0)	76 (52.1)		146 (14.8)
10 or more	83 (27.5)	219 (72.5)		302 (30.6)
**Food Insecurity and Aid**				
Severe food insecurity score (12)				
Mean (SD)	0.6 (1.5)	0.8 (1.6)	0.0357 ^c^	0.7 (1.6)
Median (Q1, Q3)	0 (0, 0)	0 (0, 1)	0.0055 ^d^	0 (0, 1)
0	385 (51.5)	362 (48.5)	0.0008 ^b^	747 (74.9)
1–3	83 (43.7)	107 (56.3)		190 (19.1)
4–6	10 (24.4)	31 (75.6)		41 (4.1)
7–9	13 (68.4)	6 (31.6)		19 (1.9)
No food insecurity	385 (51.4)	362 (48.5)	0.0124 ^b^	
Any food insecurity	106 (42.4)	144 (57.6)		
Food aid or PSNP participation (0)			<0.0001 ^b^	
No food aid or PSNP participation	372 (59.4)	254 (40.6)		626 (62.0)
Food aid (no PSNP)	42 (39.3)	65 (60.8)		107 (10.6)
PSNP (not direct support)	68 (34.0)	132 (66.0)		200 (19.8)
PSNP direct support	13 (17.1)	63 (82.9)		76 (7.5)
**Dietary Diversity**				
Minimum dietary diversity (28)			<0.0001 ^b^	
No (<= 3 food groups)	464 (47.3)	343 (35.0)		807 (82.3)
Yes (4–7 food groups)	19 (1.9)	155 (15.8)		174 (17.7)
**Total**	**495 (49.1)**	**514 (50.9)**		**1009**

^a^ For continuous variables, mean (standard deviation) and median (25th percentile, 75th percentile) are given. ^b^ *p*-value from Pearson’s chi-square test for independence. ^c^ *p*-value from independent samples *t*-test. ^d^ *p*-value from Wilcoxan rank-sum test. ^e^ Protestant, Catholic, No Religion and Other. ^f^ Wage worker, daily laborer, trader, other self-employed, retired, physically challenged, and other. ASF, animal source foods; SD, standard deviation; Q1, 25^th^ percentile; Q3, 75^th^ percentile; PSNP, Productive Safety Net Program.

**Table 2:** Adjusted odds ratios for consumption of animal source foods by religion, livelihood, and food aid or safety net participation (*n* = 1006).

	Age-Adjusted OR (95% CI)	Fully Adjusted ^a^ OR (95% CI)
Age of child—per 3-month increase	1.09 (1.04, 1.14)	1.08 (1.03, 1.13)
Religion		
Muslim	Referent group	Referent group
Orthodox	0.45 (0.35, 0.59)	0.70 (0.53, 0.94)
Other	0.44 (0.24, 0.82)	0.61 (0.31, 1.18)
Livelihood/Occupation		
Pastoral	Referent group	Referent group
Agro-pastoral	0.16 (0.10, 0.25)	0.21 (0.13, 0.35)
Agricultural/Farmer	0.10 (0.07, 0.16)	0.15 (0.09, 0.23)
Housewife and other	0.26 (0.15, 0.46)	0.32 (0.18, 0.58)
Food aid or PSNP participation		
No food aid or PSNP	Referent group	Referent group
Food aid (no PSNP)	2.26 (1.482, 3.45)	1.10 (0.68, 1.77)
PSNP (not direct support)	2.82 (2.017, 3.94)	1.74 (1.21, 2.52)
PSNP direct support	6.89 (3.71, 12.82)	4.47 (2.33, 8.56)

^a^ Fully adjusted model included age of child, religion, livelihood/occupation, and food aid or PSNP participation. PSNP, Productive Safety Net Program.
